# The Cholera

**Published:** 1885-01

**Authors:** Ad. Lippe

**Affiliations:** Philadelphia


### THE CHOLERA.

AD. LIPPE, M. D., PHILADELPHIA.

The sanitary officials and the medical profession at large are now preparing for an epidemic, which the celebrated cholera investigator, Dr. Koch, predicts will arrive in this country the coming summer. The sanitary officials devise plans to keep the cholera microbe of Koch out of the country, and the great majority of medical men are looking for a "germicide" for the cholera microbe. So far, all of Dr. Koch's scientific speculations have not hindered the progress of the cholera; and what of the cure of it? Have these microbe hunters found a microbe killer? The mortality has been, under the treatment of microbe investigators, as great as it was, before Dr. Koch presented us with his microbe. And now comes a learned medical man† who pronounces Hahnemann the original finder of the cholera microbe! We are told that in his *Lesser Writings* is to be found (p. 851) the declaration that the cause of cholera is undoubtedly an invisible cloud, "composed of, probably, millions of these miasmatic animated beings, which at first developed on the broad, marshy banks of the tepid Ganges," and "the cholera-miasm grows into an enormously increased brood of the extraordinary minute, invisible, living creatures, so inimical to human life, of which the contagious matter of the cholera must probably consist."

The deductions drawn from these declarations, our learned homœopath says, "Show that Hahnemann was a thorough believer in the cholera 'microbe,' though he had never seen it—microscopic research not having been so far advanced in 1831 as it is to-day."

*Still, camphor cannot protect the healthy from cholera, but only some preparation of copper.

† Vide HOMŒOPATHIC PHYSICIAN, Vol. IV, p. 335.

Query: Did Dr. Koch or anybody else find or expect to find the cholera microbe to be one of the miasmatic animated beings in that invisible cloud? Did any one see that invisible cloud under the microscope and in that invisible cloud a cholera microbe? What Dr. Koch investigated were not invisible clouds at all, and his discovered microbes are by no means accepted by a united microbe-hunting fraternity as exclusively belonging to cholera productions.

There are two things positively certain: 1st. Hahnemann found the curative remedies for Asiatic cholera without a microscope. He found them under the unerring law of the similars (Similia Similibus Curantur). 2d. Koch has blustered about the cholera microbe and found *no remedy* for the disease.

The only deduction we can draw from these two historical facts is, that we, as homœopathicians, can safely drop the cholera microbe, as well as the whole germ theory, as only misleading. The true healer, who fully believes in and applies understandingly the never-erring law of the similars, will cure his patients without any such theories, as did the old homœopaths.

What will be the leading homœopathic remedies in the next cholera epidemic in this country we cannot know in advance. What we do know with positive certainty are the characteristic cholera-curing symptoms of a considerable number of remedies, which by clinical experiment are verified symptoms collected in our materia medica. Unfortunately, the rising generation of so-called homœopaths have not added anything in that direction since that correct observer, Dr. Buchner, late of Munich, gave us the characteristic symptoms of Nicotin, as indicated even when collapse was imminent. Having collected all that is reliably known of the characteristic symptoms of remedies applicable for the cure of cholera, we publish them.

The patient has to be put to bed, and should be well covered with woolen blankets; he should not be permitted to rise or exert himself; mind and body should be kept in full repose and quietude.

The symptoms being fully ascertained, one or the other of the medicines best corresponding with the symptoms of the case should be administered *singly* till an improvement is perceptible, and then no more medicine should be given till the patient is worse again; if the same symptoms reappear, give the same remedy again; if the symptoms change, select another corresponding medicine. If no improvement follow, select another more appropriate remedy. *Give but one remedy at a time.* The best mode to administer the remedies is to dissolve ten pellets in half a tumblerful of clear, cold water, and to administer a teaspoonful every half hour, or if the attack be very acute, every quarter of an hour.

Camphor may be given in drop-doses of the tincture, one drop on a lump of sugar dissolved in a spoonful of water; this can be repeated every five minutes, until there is a decided mitigation of the symptoms.

A potency, the thirtieth or even a much higher one, will act quicker and more intensely curative than the crude tincture.

*Camphor.—Icy coldness of the skin.* Faintness, with pressure in the pit of the stomach; vertigo, colicky pain in the stomach; nausea, *vomiting, with cold perspiration, especially in the face;* burning in the œsophagus and stomach; cramps, especially in the calves; the upper lip is drawn up, exposing the upper teeth; eyes sunken and fixed.

*Veratrum.*—Vertigo. *Violent and profuse discharge of rice-water-like fluids upward and downward; vomiting of* FROTHY *substances;* great anguish, oppression, and spasmodic constriction of the chest; *extreme thirst for cold water in large quantities with nausea. Vomiting after drinking,* with great lassitude or diarrhœa at the same time. Distorted countenance; *cold,* pale, or *bluish face* and lips; eyes sunken and fixed, blue under the eyes, pupils contracted. Cramps in the calves, fingers, and toes; *hoarse feeble, voice,* with coldness of the mouth and tongue, dry or yellow-coated tongue. *Cold perspiration on the forehead* during the evacuations. Urinary secretions suppressed.

*Cuprum.—Ineffectual pressure to urinate, the bladder being empty. The evacuations less copious, the spasms and cramps in the stomach and chest more painful, with extreme sensitiveness to touch.* Face and lips blue and cold, voice hoarse, respiration labored, urinary secretions suppressed.

*Arsenic.—Sudden sinking of strength; burning pain* in the stomach and intestines, *restlessness,* anguish in the chest, great thirst for *cold* water, with *drinking but little at a time, vomiting as soon as he drinks.* Blueness around the sunken eyes. Face and lips blue and cold.

*Jatropha curcas.—Large watery evacuations coming away in a gush like a torrent,* with excessive vomiting of a watery substance resembling the *white of an egg. Gurgling noise in the intestines, sounding as if a bottle were emptied.* Cramps in the calves, drawing them flat.

*Secale cornutum.*—Cramps in the chest, *hands, and toes;* blue, cold, *shriveled* skin. *Aversion to heat* and being covered.

*Phosphor.*—If the thirst be excessive, *the vomiting does not*

*take place till the cold water becomes warm in the stomach*, and then the thirst is again intense. *The rice-watery evacuations contain grains like tallow. Tongue coated white.*

*Sulphur.*—Probably the most important remedy in this disease, both as prophylactic and curative medicine. *The diarrhœa commences between midnight and morning*, with or without pain, with or without vomiting, ineffectual desire to evacuate, *diarrhœa and vomiting at the same time*, numbness of the limbs, *cramps in the soles of the feet* and calves. Blueness under the eyes.

*Colchicum.*—If the least movement cause a return of vomiting, and if the nausea be accompanied by a great flow of saliva.

*Carbo veg.*—*Cold breath and tongue*, great exhaustion, *voice lost. Collapse without diarrhœa, vomiting, or spasms.* Cold perspiration on the face.

*Nicotin.*—Perfect collapse without diarrhœa or vomiting, without thirst; icy, cold perspiration on the forehead.

*China* will often restore the patient suffering from great exhaustion caused by loss of fluids.

